# Daytime Naps, Motor Memory Consolidation and Regionally Specific Sleep Spindles

**DOI:** 10.1371/journal.pone.0000341

**Published:** 2007-04-04

**Authors:** Masaki Nishida, Matthew P. Walker

**Affiliations:** Sleep and Neuroimaging Laboratory, Department of Psychiatry, Beth Israel Deaconess Medical Center, Harvard Medical School, Boston, Massachusetts, United States of America; University of Birmingham, United Kingdom

## Abstract

**Background:**

Increasing evidence demonstrates that motor-skill memories improve across a night of sleep, and that non-rapid eye movement (NREM) sleep commonly plays a role in orchestrating these consolidation enhancements. Here we show the benefit of a daytime nap on motor memory consolidation and its relationship not simply with global sleep-stage measures, but unique characteristics of sleep spindles at regionally specific locations; mapping to the corresponding memory representation.

**Methodology/Principal Findings:**

Two groups of subjects trained on a motor-skill task using their left hand – a paradigm known to result in overnight plastic changes in the contralateral, right motor cortex. Both groups trained in the morning and were tested 8 hr later, with one group obtaining a 60–90 minute intervening midday nap, while the other group remained awake. At testing, subjects that did not nap showed no significant performance improvement, yet those that did nap expressed a highly significant consolidation enhancement. Within the nap group, the amount of offline improvement showed a significant correlation with the global measure of stage-2 NREM sleep. However, topographical sleep spindle analysis revealed more precise correlations. Specifically, when spindle activity at the central electrode of the non-learning hemisphere (left) was subtracted from that in the learning hemisphere (right), representing the homeostatic difference following learning, strong positive relationships with offline memory improvement emerged–correlations that were not evident for either hemisphere alone.

**Conclusions/Significance:**

These results demonstrate that motor memories are dynamically facilitated across daytime naps, enhancements that are uniquely associated with electrophysiological events expressed at local, anatomically discrete locations of the brain.

## Introduction

A growing corpus of literature continues to demonstrate that, following learning, additional “offline” memory improvements develop during sleep [1], [2]. Evidence of sleep-dependent consolidation now exists across numerous memory domains, including procedural as well as declarative memory [3]. Regarding procedural motor memory, several studies have demonstrated that the extent of initial learning, and the subsequent offline enhancement, commonly correlate with non-rapid eye movement (NREM) sleep, and neurophysiological characteristics of NREM [4]–[8], although see [9]. For example, it has been shown that offline motor-memory enhancements specifically develop across a night of sleep, with the extent of improvement demonstrating a positive relationship with the amount of stage-2 NREM sleep, especially in the last quarter of the night [4]. Considering sleep spindles – a defining electrophysiological signature of NREM involving short (∼1 ) synchronous burst of activity (12–15 Hz) – may represent candidate triggers of synaptic potentiation leading to neural plasticity [10]–[12], and that spindle activity is highest late in the night [13], this latter correlation was hypothesized to reflected an association between spindle activity and offline memory improvement [4].

At a neural level, recent functional imaging data have also demonstrated that these overnight motor memory improvements are associated with a systems-level, plastic reorganization within the brain, including a lateralized expansion and increased activation in the right primary motor cortex; contralateral to the hand (left) learning the motor skill memory [14]. While sleep stage correlations represent a global measure of association with memory enhancement, such neuroimaging data illustrate that sleep produces highly localized changes in discrete brain circuits. Therefore, if sleep and specific electrophysiological characteristics of sleep are contributing to these circuit changes, then topographical EEG examination should reveal more selective, local associations with memory improvement.

Here we investigate the relationship between regionally specific sleep spindle activity and motor memory consolidation, using a nap paradigm, incorporating the above described motor-sequence task. The advantage of this design is twofold; a) the offline motor-skill enhancements are associated with a localized plastic change in the contralateral motor cortex, situated proximal to standard EEG recording sites where sleep spindles are commonly expressed [15], and b) it allows a within subject comparison of spindle activity between the predominant “learning” (right) hemisphere relative to the “non-learning” (left) hemisphere. We use the terms “learning” and “non-learning” hemispheres simply to reflect the known lateralized, offline plastic changes observe across a night of sleep using this task [14]; although it should be noted that practice-dependent motor learning using the non-dominant hand often involves bilateral motor cortex activation (e.g. [16]). We tested the hypothesis that i) daytime naps would result in significant offline learning enhancements of motor-skill memory, and ii) the magnitude of enhancement would not only be proportional to the amount of stage-2 NREM and the extent of sleep spindle activity, but specifically with spindle activity in the hemisphere associated with offline learning (right), *relative* to homeostatic/non-task relevant spindle activity in the “non-learning” hemisphere (left).

## Results

**Figure 1:**
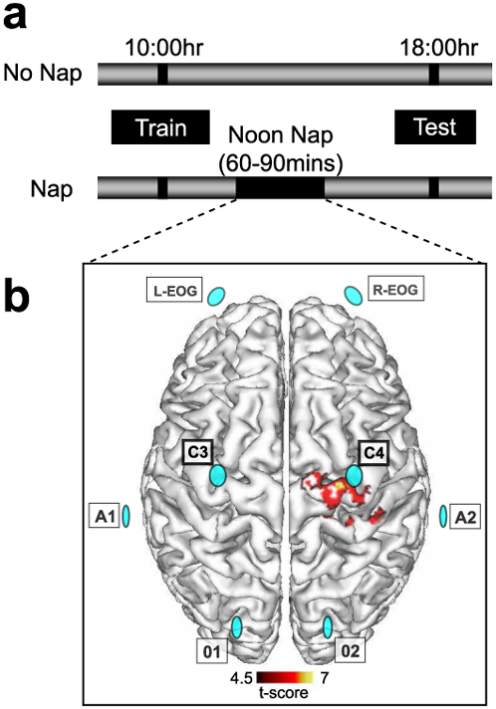
Experimental design. a, Both groups were trained in the morning and test 8 hr later. Following training, the Nap group obtained a 60–90 min midday sleep period, while the No nap group remained awake across the 8hr delay. b, The nap period was recorded with digitized polysomnography (PSG) using a referenced electrode montage. The electrode montage (represented by blue discs) included EEG sites C3 and C4, covering localized learning regions of interest (motor cortex), together with O1 and O2 sites (referenced to A1 and A2, left and right outer canthi). A bipolar left and right submental array was used for monitoring of EMG (not shown), while left and right EOG channels (L-EOG, R-EOG) were used for eye-movement evaluation. For reference purposes, the electrode array is superimposed on top of the known fMRI changes in activation that occur across a night of sleep (modified from [14]; EEG anatomical precision not inferred), demonstrating enhanced activation in the right, contralateral motor cortex (activation strength in red/yellow, display threshold; *P*<0.05^FWE^).

Two groups of subjects trained on a motor-skill task using their left hand–a paradigm known to result in overnight plastic changes in the contralateral, right motor cortex. Both groups trained in the morning and were tested 8 hr later, with one group obtaining a 60–90 minute intervening midday nap, while the other group remained awake (Figure 1).

### Behavioural performance

#### Practice-dependent learning

Across the training session, performance speed improved in both the nap and non-nap groups, without loss of accuracy. Comparing initial baseline (average of the first 2 trials of training) to post-training performance, subjects in the no-nap group demonstrated an average improvement of 5.37 seq/trial (baseline: 16.6 seq/trial, post-training: 22.0 seq/trial; paired t-test t_(11)_ = 4.43, p = 0.001). Similarly in the nap group, a significant average improvement of 6.42 seq/trial was achieved across training (baseline: 17.0 seq/trial, post-training: 23.4 seq/trial; paired t-test t_(13)_ = 4.78, p<0.001). No significant change in performance accuracy occurred across the training session in either the no-nap group (baseline: 0.25 errors/seq, post-training: 0.21 seq/trial) or nap group (baseline: 0.28 seq/trial, post-training: 0.21 seq/trial), both p>0.49. A comparison of training performance between the nap and no-nap groups revealed no significant difference, for speed or accuracy, at baseline, post-training, or in the amount of improvement across training (unpaired t-test; all p>0.48). Therefore, both groups similarly acquired the motor skill memory across the training session.

#### Offline, practice-independent learning

**Figure 2:**
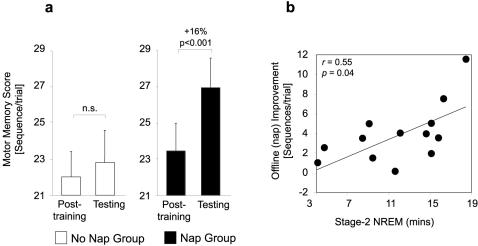
Motor memory performance. a, Motor skill performance at the end of the initial training session (“post-training”) compared with later testing following the 8hr intervening period in the No Nap and Nap groups. b, Correlation between the extent of offline memory improvement and the amount of stage-2 NREM sleep obtained within the Nap group.

Consistent with previous findings [4], [9], [17]–[19], there was no evidence of delayed offline memory enhancement across the day in those subjects that remained awake. Specifically, performance in the no-nap group changed from 22.0 seq/trial (post-training) to 22.8 seq/trial (test; paired t-test t_(11)_ = 0.80, p = 0.44), representing a non-significant 3.8% (0.8 seq/trial) increase at later testing (p = 0.29; Figure 2a).

In contrast, subjects in the nap group demonstrated a highly significant 16% consolidation enhancement (3.5 seq/trial, p = 0.002), improving from 23.4 seq/trial (post-training) to 27.0 seq/trial (test; paired t-test t_(13)_ = 4.05, p = 0.001; Figure 2b). Furthermore, the magnitude of offline enhancement in the nap group was larger than that observed in the no-nap group (unpaired t-test t_(24)_ = 1.99, p = 0.058). As with training, there were no significant differences in performance error within or between groups across the delay period (all p>0.20), indicating that no loss of accuracy accompanied these improvements in motor sequence production.

Therefore, no delayed improvements were observed in subjects that remained awake across the day, while those that obtain a midday nap expressed significant offline consolidation enhancements across the day.

### Sleep stage analysis

Sleep stage polysomnography (PSG) characteristics for the nap period (nap group) are summarized in Table 1, with an average nap time of 67 minutes and a predominance of NREM (combined stages 1–4; 79%) over REM (14%).

**Table 1:** Amount of sleep time and percentage spent in each sleep stage of nap group (mean±SEM)

	Sleep time	Percentage
Total nap time	66.06±4.48	
Sleep efficiency	77.13±4.98 (%)	
Stage 2 latency	5.24±0.78	
REM latency	37.49±6.88	
Stage 1	10.72±1.71	17.96±3.60%
Stage 2	11.98±1.15	18.25±1.61%
SWS	28.78±3.48	43.60±4.98%
REM	10.50±2.44	13.86±2.96%

Mean duration (in minutes) and standard error (SEM) of total nap time and sleep stages, together with REM latency. SWS, slow wave sleep (Stage 3 and Stage 4); REM, rapid eye movement sleep; Sleep efficiency, (total sleep time/total time in bed)×100.

To examine our experimental hypothesis, we correlated the amount of stage-2 NREM sleep with the amount of offline memory improvement across the nap group. As demonstrated in Figure 2b, there was a significant positive relationship between stage-2 NREM and the amount of motor skill enhancement (Pearson correlation *r* = 55, p = 0.04), with those subjects obtaining the most stage-2 NREM demonstrating the largest consolidation benefit at later testing.

Also as expected, no correlations were evident between performance improvement and other sleep stages (stage-1 NREM, SWS or REM, all *r*<0.14). Therefore, at a global level of sleep analysis, there was a positive relationship between nap-related memory improvement, and stage-2 NREM, similar to that reported across a night of sleep.

### Sleep spindle analysis

One participant was excluded from the analysis due to abnormal spindle activity, representing density values over three standard deviations above the group mean (although it should be noted that if included, this participant increased the below described strength of correlations). Spindle analysis focused on *a priori* central EEG sites C3 and C4, and specifically the difference between activity in the learning and non-learning hemispheres ([C4–C3]).

**Figure 3:**
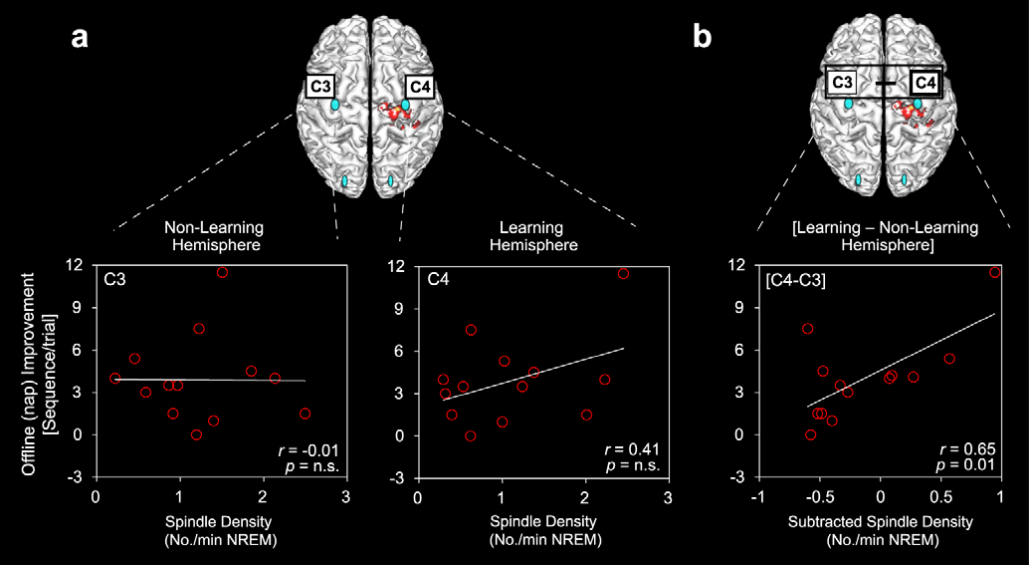
Spindle density and offline (nap) memory enhancement. a, Correlations between offline motor memory enhancement and spindle density in the non-learning hemisphere (electrode site C3) and learning hemisphere (electrode site C4) individually. b, Correlations between offline motor memory improvement and the subtracted difference in spindle density between the learning hemisphere versus the non-learning hemisphere (C4–C3). Pearson's correlation coefficients (r) and corresponding significance (p) are displayed within each correlation window.

When first considering each central electrode site separately, there was no significant difference in spindle density between the learning versus non-learning hemisphere (paired t-test t_(12)_ = 0.98, p = 0.35). Furthermore, there was no correlation between offline memory improvement and spindle density at either C3 or C4 electrode sites individually (Figure 3a; both *r*<0.41). However, when spindle density in the non-learning hemisphere (electrode C3) was subtracted from the learning hemisphere (electrode C4), representing the homeostatic difference following learning ([C4–C3]), a significant positive correlation was identified between the amount of offline motor skill improvement across the nap and the density of spindles (*r* = 65, p = 0.01; Figure 3c). In order to determine whether these correlations were locally specific to the central electrode sites proximal to the motor cortex region of interest, rather than a more general hemispheric difference or asymmetry, we repeated these correlations using spindle activity from posterior occipital electrodes. No significant relationships were evident for either of the occipital electrode sites alone (O3 or O4), or for the subtracted difference ([O4–O3]; all *r*<0.29, p>0.37); indicating that the above correlations represented a regionally specific spindle association with offline learning in central locations.

**Figure 4:**
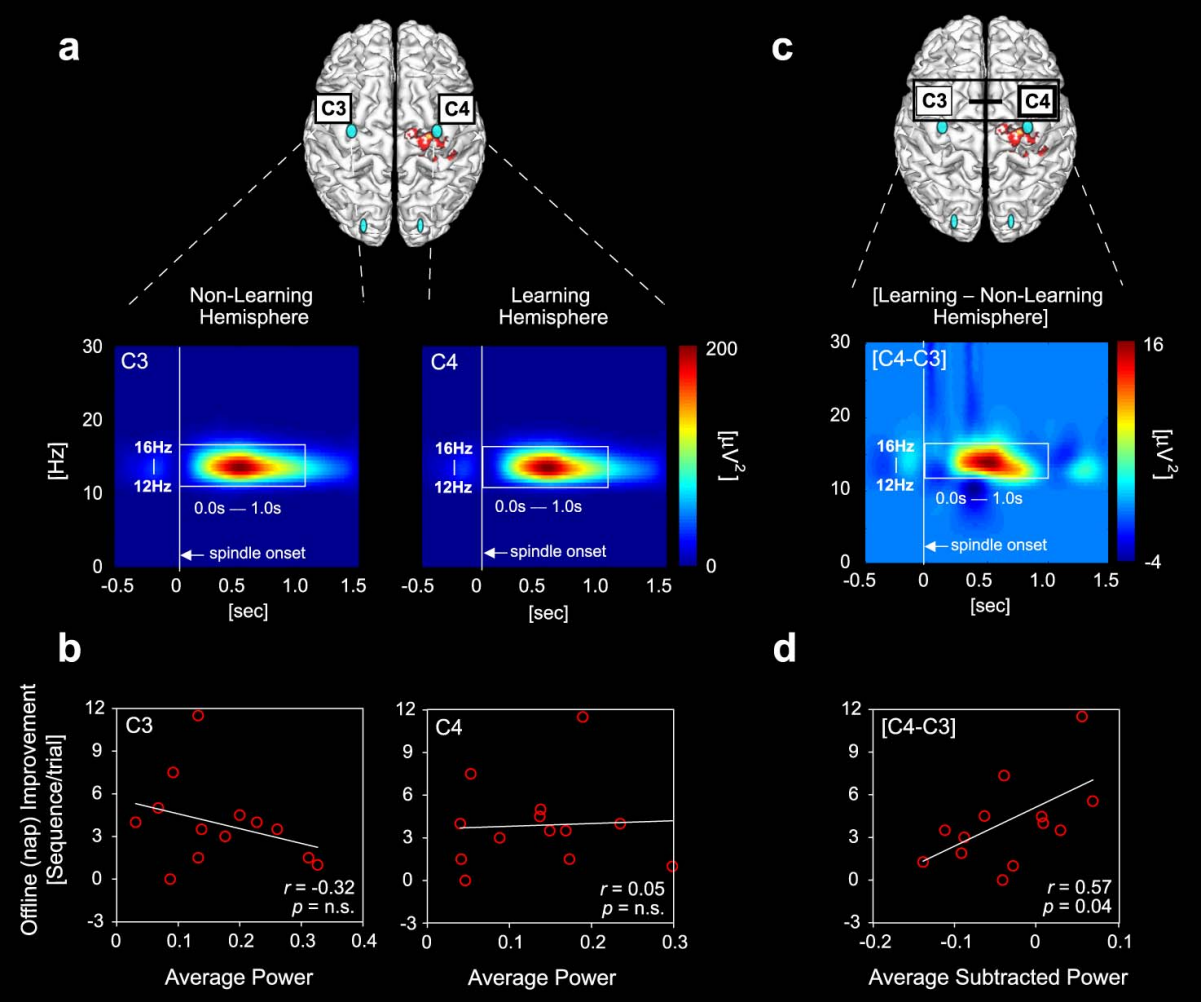
Spindle power and offline motor memory enhancement. a, Spindle event-related time-frequency activity, evaluated across a 2 second epoch (0.5 seconds before spindle onset and the 1.5 seconds after), incorporating a frequency range of 1–30 Hz after band-pass filtering (12–16 Hz, encompassing sigma-band power), in the non-learning hemisphere (electrode site C3) and learning hemisphere (electrode site C4) individually. b, Corresponding correlations between motor memory improvement and mean spindle power. c, Subtracted difference in spindle power between the learning hemisphere versus the non-learning hemisphere (C4–C3). d, Corresponding correlation between motor memory improvement and subtracted spindle power (C4–C3). Spindle power is depicted in µV^2^ (strength indicated by right side color bar). Pearson's correlation coefficients (*r*) and corresponding significance (*p*) are displayed within each correlation window.

As with spindle density, spindle power analysis revealed similar locally specific correlations with over-nap improvement (Figure 4). First, no significant differences were observed between the amount of spindle power at C3 (non-learning hemisphere) compared to C4 (learning hemisphere) electrodes (paired t-test t_(12)_ = 1.81, p = 0.10). Furthermore, for each electrode site separately, no significant correlation was observed between the amount of offline memory improvement and the magnitude of spindle power (Figure 4b; *r*<0.32). In contrast, however, when spindle power in the non-learning hemisphere at electrode C3 was subtracted from that in the learning hemisphere at electrode site C4, a strong and significant correlation with offline memory enhancement was revealed (*r* = 0.57, p = 0.04; Figure 4d). There was once again no significant relationships between spindle power in occipital electrode sites and offline improvement (either O3 or O4 alone, or for the subtracted difference [O4-O3]; all *r*<0.25, p>0.44); further confirming the local specificity of association between spindle power and offline learning in central regions.

Therefore, by subtracting non-specific (to this task) spindle activity in the central non-learning hemisphere (C3) from that recorded in the learning hemisphere (C4), a clear predictive relationship was evident between the density and power of locally expressed spindles and the amount of offline memory enhancement across the nap episode.

It may, however, be possible that these predictive correlations reflect use-dependent differences between hemispheres, triggered by the daytime training session, and not necessarily offline learning-dependent associations (i.e. spindle asymmetry may be driven by increased hand-use prior to the nap, produced by the initial practice session). To further investigate this possibility, we correlated spindle activity with the total number of key-strokes accomplish across the 12 trials of training, independent of being correct or incorrect; reflecting the aggregate of digit use. Neither subtracted (C4–C3) spindle frequency or power demonstrated a significant relationship with the total number of key-strokes achieved during training (*r* = 0.35, p = 0.24; *r* = 0.42, p = 0.15, respectively). Therefore, a use-dependent explanation for the significant correlations reported in Figures 3 and 4 appears inadequate.

## Discussion

While it is known that motor skill memories improve offline across a night sleep, and can correlate with global sleep-stage measures, using a nap paradigm, here we demonstrate that offline motor memory enhancement is proportional not simply with basic sleep stages, but with locally constrained increases in sleep-spindle activity in central regions of the learning hemisphere, relative to more non-specific activity in the non-learning hemisphere. These findings offer more precise insights into the nature of sleep-dependent memory consolidation at an anatomical, physiological, and behavioral level, each of which we now focus on.

Although a general correlation was observed between memory improvement and the amount of stage-2 NREM sleep–a global measure of brain physiology–more detailed topographical analyses identified significant relationships with regionally specific sleep-spindles at a local level of brain anatomy. It should perhaps not be surprising that, if a memory representation is manifest in discrete neural circuits of the brain, then brain-states capable of modulating them offline will likewise operate at a similarly sophisticated local, anatomically specific level. Supporting this local sleep hypothesis, our results build on a growing body of evidence indicating that daytime waking experience can trigger regionally specific modifications in post-training sleep complexion [20]–[22], and indicate that a similar local sleep change may also operate in the facilitation of offline consolidation and plasticity, leading to post-sleep memory improvements.

Pioneering work by Tononi and colleagues has demonstrated that overnight memory enhancements are regulated by homeostatic, regionally specific changes in sleep physiology [23]. For example, following training on a motor reaching task, a corresponding increase in subsequent NREM slow-wave activity (SWA) was observed in parietal regions known to represent such memories, with the amount of SWA increase being proportional both to the extent of initial daytime learning and the degree of next-day memory improvement [7]. Conversely, motor limb inactivation through arm immobilization results in a corresponding decrease of SWA in localized sensori-motor regions [24].

Our findings add to this concept of local sleep modulation by demonstrating that a phasic electrophysiological event–sleep spindles–also displays a regionally specific association with offline memory enhancement. However, this change was only revealed when subtracting non-specific spindle activity in the non-learning hemisphere from that measured in the learning hemisphere, indicating that such a change is subtle. This too should not be surprising considering that the daytime learning experience only lasted 12 minutes. Nevertheless, when this method was implemented, a clear predictive relationship emerged between residual, local spindle activity in the learning hemisphere and the extent of offline consolidation improvement. Such within subject (between hemisphere) subtractions appear to provide a sensitive means of extracting learning-dependent signal in brain activity; a relationship that would likely be lost at a between-subjects level.

A difference of interpretation between our study findings and those of Tononi and colleagues concerns the proposed function of such local sleep changes. The above described increases in regional SWA are hypothesized to represent processes that reduce or depress synaptic plasticity following daytime experience, thereby preventing the circumstance of over potentiated networks the following day [23]. In contrast, since sleep spindles have been proposed as a neurophysiological marker of synaptic potentiation–with corresponding electrophysiological frequencies more commonly associated with long-term potentiation (LTP) than de-potentiation (LTD) – we suggest that the local effects of increased spindle activity in the learning-related hemisphere represents the *facilitation* of intrinsic synaptic plasticity, not its diminution. We do not, however, feel that these two hypotheses are mutually exclusive, nor diametrically opposed. Instead, we entertain that they may occur in a co-operative, symbiotic manner across a night of sleep (SWA dominating early in the night, spindle activity most dominant late in the night), and in the endeavour of refining and subsequently enhancing recently formed memory representations.

At the global sleep-stage level, we replicate findings of our own, and those of others [4]–[8], indicating that consolidation of basic motor skills are preferentially associated with NREM sleep, here stage-2 NREM across a daytime nap (although see [25] for discussion of task difficulty and REM- versus NREM-dependency). These selective sleep-stage correlations indicate that it is not simply obtaining any period of behavioral quiescence (representing a passive time of minimal sensori-motor interference) that favours consolidation, since it was a specific stage of sleep that predicted offline memory enhancement, not total sleep (inactivity) time. In fact, previous motor skill studies have shown that periods of daytime wake with learning-effector immobilization (negating corresponding limb interference, hence offering the opportunity for consolidation), not only result in the absence of any memory improvement, but can produce learning deteriorations [4], [24].

At a behavioral level, our demonstration of offline-memory enhancement across a nap is consistent with previous work demonstrating similar daytime sleep benefits for both sensory-perceptual [26], as well as episodic declarative memory [27]. Moreover, the magnitude of enhancement we observed across the nap period was similar to the amount of improvement normally expressed following an entire night of sleep [4], [14], [17], [18], [28]. One interpretation of this finding is that daytime “power” naps trigger a form of accelerated consolidation, leading to more rapid offline memory improvements. Alternatively, and the hypothesis we subscribe to, it may be that an entire night of sleep contains multiple sleep-stage windows preferentially devoted to the consolidation of many different forms of daytime learning [29]. As a consequence, for any one specific memory, an entire night of sleep is not necessary, only the corresponding specific sleep state/window.

In summary, here we demonstrate that motor memories are dynamically facilitated across daytime naps; improvements that are not simply associated with a particular stage of sleep, but with unique electrophysiological events at anatomically discrete locations of the human brain. These findings are compatible with the notion of a homeostatic response by the sleeping brain to neuroplastic demands; the goal of which is to sculpt the most efficient neural representation of recently acquired information.

## Materials and Methods

### Participants

A total of 26 healthy right-handed subjects between the ages of 18 and 30 were assigned to either a nap group (*n* = 14; 7 males, mean age 24.3 [S.D.±2.0]) or no-nap group (*n* = 12, 8 males, mean age 23.1 [S.D.±1.4]). Subjects had no prior history of drug or alcohol abuse, neurological, psychiatric or sleep disorders, were maintaining a regular sleep schedule 1 week prior to the study, as measured using sleep logs. Subjects were also required to abstain from caffeine and alcohol throughout the course of the study, and also refrain from non-experimentally measured naps, confirmed by post-experimental questionnaire. The study was approved by the local human studies committee and conducted according to the principles expressed in the Declaration of Helsinki, with all subjects provided written informed consent.

### Motor Skill Task

The sequential finger-tapping task required subjects to press four numeric keys on a standard computer keyboard with the fingers of their left (non-dominant) hand, repeating the five element sequence, 4-1-3-2-4, “as quickly and as accurately as possible” for a period of 30 seconds (for details, see [4], [17]). The initial training session consisted of twelve 30 second trials with 30 second rest periods between trials, and lasted a total of 12 minutes. Trials were automatically scored for both performance speed (number of correctly typed sequences per trial) and accuracy (error rate: number of errors per sequence). Performance on the first two trials of the training session were taken as the “baseline” measure, while averaged scores from the final three trials were defined as “post-training” performance. At the subsequent “test”, subjects performed three 30 second trials of the same sequence, separated by 30 second rest periods, with the scores again being averaged. Offline memory consolidation (practice-independent) improvement was defined as the difference between post-training performance and subsequent test performance [4], [17].

### Experimental design: Nap and non-nap groups

Both groups trained on the motor skill task at 10AM and, eights hours later, were tested on the task at 6PM (Figure 1a). Standard daily activities were conducted by participants between training and testing, except that in the nap group, subjects undertook a 60–90 min sleep period at midday, monitored using polysomnography (PSG). The no-nap group was instructed not to nap during the day, with confirmation obtained by post-experimental questionnaire. Similarly, beyond the experimentally recorded midday nap, those in the nap group were also instructed not to nap before or after the noon sleep session, also confirmed by post-experimental questionnaire. PSG recording was performed in accordance with standardized techniques [30], using digital EEG, EMG and EOG signals acquired with a Grass Colleague system (sampling rate: 100 Hz, high- and low-pass filter 0.3 Hz and 35 Hz respectively, notch filter 60 Hz). A referenced PSG electrode montage was utilized, including EEG sites C3 and C4 (referenced to A1 and A2, left and right outer canthi), proximal to the localized learning region of interest (motor cortex; [14]; Figure 1b).

### PSG and EEG analysis

Each sleep epoch of the PSG record was scored according to standard criteria [30], blind to subjects behavioral task performance. The signals were displayed on a computer monitor and rated visually, epoch by epoch, as either NREM stages 1–4, rapid eye movement (REM) sleep, awake or movement time. Slow wave sleep (SWS) consisted of stage 3 and stage 4 NREM sleep.

Upon removal of waking epochs and movement/muscle artefacts from sleep recordings, sleep spindles analysis focused on NREM sleep epochs, at all electrodes sites, using an automatic algorithm in Matlab (The MathWorks Inc, Natick, MA). The raw EEG was first band-pass filtered between 12 and 16 Hz using a linear finite impulse response (FIR) filter (EEGLAB toolbox [http://www.sccn.ucsd.edu/eeglab/]).

Analyses focused on two spindle characteristics potentially influencing plasticity–amount (density) and strength (spectral power) [12]. Spindle density was evaluated using two complementary methods. The first involved visual scoring, calculated as the mean number of spindles per minute of NREM sleep. The second involved the application of a dynamic, automated EEG spindle detection algorithm, developed by Huber, Ferrarelli, Tononi and colleagues [31]. In short (but for details see [31]), the amplitude of the rectified signal was used as a unique time series, identifying amplitude fluctuations exceeding threshold values, with the lower and upper values set at two and eight times the average amplitude. The peak amplitude for each spindle was defined as the local maximum above the threshold, with the beginning and end of the spindle defined as points immediately preceding or following this peak, when the amplitude of the time series dropped below the cut-off threshold. As with visual scoring, automated spindle density was calculated as the mean number of spindles per minute epoch of NREM sleep. Based on the high specificity and sensitivity [31], and its standardized applicability for this and future studies, automated spindle detection was used as our experimental measure of choice. It should be recognized that the sensitivity and specificity of this technique for identifying spindle activity across waking epochs, such as in the no-nap group, has not been determined, although the occurrence of waking spindling is usually indicative of pathological disease, hence would not be expected [32].

Quantification of sleep spindle spectral power was also determined using the method of Huber, Ferrarelli, Tononi and colleagues (for details, see [31]), similarly implemented in Matlab. In brief, event-related time-frequency activity was evaluated in 2 second epochs (0.5 seconds before spindle onset and the 1.5 seconds after) and for a frequency range of 1–30 Hz after band-pass filtering (12–16 Hz), encompassing sigma-band power. The time-frequency representations were calculated for all spindle events of each subject by using the Morlet wavelet transform to compute the spectral power. Detailed methods of the wavelet analysis are available as Supplemental Text S1. The averaged sigma power value was calculated between a time range from spindle onset to 1.0 sec, and a frequency range of 12 Hz to 16 Hz.

### Statistical Analysis

Analyses were carried out using paired and two-sample two-tailed Student's t-test, together with Pearson's correlation coefficients.

## Supporting Information

Text S1 Wavelet analysis
(0.03 MB DOC)
