# Supplementary material for: Daytime Naps, Motor Memory Consolidation and Regionally Specific Sleep Spindles
Source: PLoS One. 2007 Apr 4;2(4):e341. doi: 10.1371/journal.pone.0000341 (PMC1828623; doi:10.1371/journal.pone.0000341)
Supplement: Text S1 — Wavelet analysis (0.03 MB DOC) [file pone.0000341.s001.doc]

**Wavelet analysis**

To quantify the spectral power of sleep spindles, event-related time-frequency activity was evaluated in 2 second epochs (0.5 seconds before spindle onset and the 1.5 seconds after) and for a frequency range of 1-30 Hz after band-pass filtering (12-16 Hz), encompassing sigma-band power, using Morlet wavelet transformations, also implemented in Matlab. For the wavelet transformation, a complex Morlet mother function was used [1,2]. The energy $S(t,f)$ at frequency *f* and time *t* is given by the squared norm of the convolution of the Morlet wavelet with the EEG signal $\nu(t)$

$S(t,f)=\left|w(t,f)\times\nu(t)\right|^{2}$,

where the Morlet wavelet, $w(t,f)$ is defined by

$$\text{w(t,f) }=\text{ }\sqrt{\pi}\sigma_{b}\text{exp }\left(\text{-}\left(\frac{\text{t}}{\sigma_{\text{b}}}\right)^{2}\right)\text{ }\times\text{ exp(i2}\pi f\text{t) .}$$

with $\sigma_{b}$being the bandwidth parameter, and the ‘width’ of the wavelet $m=2\pi\sigma_{b}f$ set to a value 7. The time-frequency representations were calculated for all spindle events of each subject and then averaged. In addition, the averaged sigma power value of each subject was also calculated between a time range from spindle onset to 1.0 sec, and a frequency range of 12 Hz to 16 Hz.

1. Jensen O, Tesche CD (2002) Frontal theta activity in humans increases with memory load in a working memory task. Eur J Neurosci 15: 1395-1399.

2. Kronland-Martinet R, Morlet J, Grossman A (1987) Analysis of Sound Patterns Through Wavelet Transforms. International Journal of Pattern Recognition and Artificial Intelligence 1: 273-302.
